# Recovery of Dysphagia in Lateral Medullary Stroke

**DOI:** 10.1155/2014/404871

**Published:** 2014-06-17

**Authors:** Hitesh Gupta, Alakananda Banerjee

**Affiliations:** Max Healthcare, Saket, New Delhi 110017, India

## Abstract

Lateral medullary stroke is typically associated with increased likelihood of occurrence of dysphagia and exhibits the most severe and persistent form. Worldwide little research exists on dysphagia in brainstem stroke. An estimated 15% of all patients admitted to stroke rehabilitation units experience a brainstem stroke out of which about 47% suffer from dysphagia. In India, a study showed that 22.3% of posterior circulation stroke patients develop dysphagia. Dearth of literature on dysphagia and its outcome in brainstem stroke particularly lateral medullary stroke motivated the author to present an actual case study of a patient who had dysphagia following a lateral medullary infarct. This paper documents the severity and management approach of dysphagia in brainstem stroke, with traditional dysphagia therapy and VitalStim therapy. Despite being diagnosed with a severe form of dysphagia followed by late treatment intervention, the patient had complete recovery of the swallowing function.

## 1. Introduction

Swallowing mechanism is a sequential event of oral, pharyngeal, and esophageal phases that transports saliva, ingested solids, and fluid from mouth to the stomach and protects the airways during swallowing. Pharyngeal function involves numerous interacting control mechanisms that ultimately link pharyngeal contraction patterns to the adjacent oral cavity and esophagus.

Dysphagia (difficulty in eating and swallowing) is extremely common following a brainstem stroke. About 15% of all patients admitted to stroke rehabilitation units experience a brainstem stroke out of which about 40%–47% of the patients suffer from dysphagia [1]. Dysphagia following brainstem stroke is characteristically associated with a greater likelihood of occurrence, showing signs of most severe form of dysphagia, compared to hemispheric strokes [2]. Worldwide little research exists on dysphagia in brainstem stroke.

Dysphagia has been associated with respiratory complications, increased risk of aspiration pneumonia, nutritional compromise, and dehydration. It is also socially penalizing and affects the patient's quality of life.

A brainstem stroke affects the swallowing function as the major swallowing centers of the nucleus tractus solitarius (NTS), nucleus ambiguus (NA), and the reticular formation are situated in the dorsolateral medulla oblongata [3]. As a result, dysphagia following a lateral medullary stroke (LMS) is often more severe and spontaneous recovery may not completely restore the swallowing function. It may persist for life time or may take months or years to resolve [2]. Factors, such as lesion size and actual stroke location, as detected and correlated with brainstem MRI, can play a significant role in determining dysphagia morbidity. However, MRI results can demonstrate only the area of the infarct and not the swallowing-related structures and, therefore, cannot clarify the variation of swallowing disorders among patients with LMS [3].

Treatment for dysphagia involves traditional therapy [4, 5] (diet modification, exercises to strengthen the oropharyngeal musculature, compensatory maneuvers to facilitate laryngeal elevation and closure during swallowing, and techniques to stimulate and strengthen the swallowing reflex) and advanced VitalStim therapy.

VitalStim therapy is a special form of neuromuscular electrical stimulation (NMES) device, which has received FDA clearance to be used for treatment of pharyngeal dysphagia. It can only be administered under the direction of certified healthcare professionals (speech language pathologists and occupational therapists) [6]. On direct electrical stimulation, contraction of swallowing muscles occurs, which is due to the depolarization of peripheral motor nerve after acetylcholine is released at the end plates.

VitalStim is a dual-channel electrotherapy system, which uses small calibrated electrical current delivered by specially designed electrodes to stimulate the muscles responsible for swallowing. At the same time, trained specialists help patients “reeducate” their muscles through rehabilitation therapy.

Electrodes are simultaneously activated over the submental and laryngeal regions on the throat with the aim of producing a simultaneous contraction of the mylohyoid muscle in the submental region (to elevate the hyoid bone) and the thyrohyoid muscle in the neck (to elevate larynx to the hyoid bone). In VitalStim therapy, electrode placement varies if there is

- insufficient length of the neck;
- fresh surgical incision (no direct placement);
- presence of indwelling foreign materials like tracheostomy, staples, sutures, and so forth (avoid placement over them).

VitalStim therapy is contraindicated if patient is not conscious, uncooperative, has behavioral issues, or has any implants like pacemaker and so forth [6].

VitalStim therapy has been used to reeducate patients to utilize their pharyngeal muscles in the throat for patterned activity to commence or reestablish swallowing [4]. It has been theorized that this small electrical stimulation may aid swallowing either by augmenting hyolaryngeal elevation or by escalating sensory input into the central nervous system. The current stimulates motor nerves in the throat instigating the muscles responsible for swallowing to contract. The quality of the swallowing function improves and with repetition muscles may be reeducated. This case study aims to document the severity and the advanced technological management of dysphagia in lateral medullary infarct.

## 2. Case Study

The patient, a 29-year-old male, came to the Department of Physiotherapy and Rehabilitation, Max Super Speciality Hospital, Saket, New Delhi, with the complaint of inability to swallow. He had to spit out saliva frequently as he was unable to swallow it. The patient had a history of right medullary stroke (infarction) 1 year back. He was treated in a local hospital in Punjab. During the course of his hospitalization, he acquired severe pneumonia due to aspiration while he was feeding orally. Therefore, a nasogastric tube (NGT) was placed for nutritional support. The length of stay in the hospital was two months where all of his functions recovered except swallowing. The patient was discharged with the NGT. He was sent to a neurologist for examination. Clinical dysphagia evaluation [5] was done by a dysphagia therapist, which included a detailed history of the subjective complaints and medical status, cranial nerve testing, and an examination of the phases of swallowing. Cervical auscultation and respiratory status examination were also done [7]. Food trial was not done as the patient could not swallow. It was observed that the patient could not swallow his saliva.

The dysphagia assessment revealed that the patient had good oral motor functions and was able to chew and propel bolus from the mouth but was unable to swallow. The impairment was found in the pharyngeal phase. No hyolaryngeal movement was elicited in an attempt to swallow voluntarily or on stimulating swallowing reflex indicating a complete loss of pharyngeal swallowing phase. This can be explained as follows: when mediated by the swallowing center in the medulla, as any stimulus reaches the pharynx, it causes the food to be pushed further back into pharynx and esophagus by rhythmic but involuntary contractions of several muscles in the back of the mouth, pharynx, and esophagus. Laryngeal elevation is essential for airway protection during the pharyngeal phase of swallowing. It aids opposition of the arytenoids to the base of epiglottis for laryngeal vestibule closure and helps shape the aryepiglottic sinuses to divert the bolus laterally around the vestibule. Anterior movement of the hyoid in conjunction with laryngeal elevation helps to pull open the flexed upper esophageal sphincter so that food or liquid may be propelled through it and into the esophagus [6].

During phonation, soft palate movement was reduced, but protective reflexes like gag, cough, and the ability to clear throat were good. Among the lower cranial nerves, glossopharyngeal (IX nerve) and vagus (X nerve) nerves were found to be relatively more impaired. Disuse atrophy made his chance of recovery even worse as it is believed that, after 72 hours of stroke, disuse atrophy starts if any particular muscle does not work [8–10]. In this case his pharyngeal muscles were not in use for more than a year.

American Speech-Language-Hearing Association (ASHA) National Outcome Measurement System (NOMS) swallowing level scale was used as a parameter for evaluating the swallowing function on which the patient was rated level 1. The ASHA level is a measurement of both the level of supervision required and the diet level that intuitively reflects a patient's functional status [11]. A FEES (fibreoptic endoscopy evaluation of swallowing) was not done.

## 3. Course of Treatment Planning and Recovery

After detailed assessment and counseling of the patient, a treatment program of traditional therapy combined with VitalStim therapy was planned in the outpatient department of physiotherapy and rehabilitation to be followed five days a week. Studies have shown the effectiveness of combined traditional therapy and VitalStim therapy in stroke patients [6, 12]. The treatment was started and, for convenience, the management was divided into two phases.

## 4. Phase One

The initial goal was to elicit swallowing reflex to initiate hyolaryngeal movement. Documented laryngeal elevation in normal adults is approximately 0–2.50 cm or 20 mm [13, 14]. Therapy commenced after familiarizing the patient with the treatment protocol and VitalStim device.

The sensory threshold of the patient was identified as the lowest current level at which he reported a “tingling sensation” on the skin. Following this, the current intensity was raised to the level reported as “grabbing sensation” by the patient, which in our case ranged between 12 and 14 mA.

Each VitalStim therapy session lasted for 60 minutes and five sessions were taken per week. In conjunction with VitalStim therapy, traditional therapy was provided which involved sensory stimulatory activities to elicit swallowing reflex with the use of lemon drops, salt, honey, and ice under the supervision of the dysphagia therapist. Sensory input from the gut has a major influence on the activity of brainstem swallowing centers and cortical sensory and motor areas [15].

A flicker in the hyolaryngeal excursion on stimulating swallowing reflex was noticed on clinical palpations after completion of 34 sessions of VitalStim therapy. Subjectively the patient was sensitive to voluntary swallowing attempts, though after every attempt, it was seen that there were clinical laryngeal penetration signs like coughing and wet and gurgling voice which required throat clearing followed by spitting of saliva. Pharyngeal strengthening exercises like Shaker exercise, Mendelssohn's maneuver, effortful swallow, and Masako exercise were also incorporated [5, 16].

## 5. Phase Two

After 12 weeks, approximately more than 50% of hyolaryngeal excursion, not functional (MMT) [14], was observed on clinical palpation. Food trials were introduced along with VitalStim therapy. Among the eight types of consistencies of food groups [5], initially group 4 (pudding thick consistency) foods like ice cream, curd, and jelly were given as they have minimal risk of aspiration. Patients with neurogenic dysphagia experience more difficulty with fluids than with thicker or solid consistencies [17].

The patient experienced weakness of pharyngeal peristalsis while swallowing, resulting in food residue in the pharyngeal pockets followed by throat clearing and coughing. The patient had coughs during food trials but was gradually able to swallow the food bolus partially along with the saliva by attempting to swallow repeatedly after throat clearing instead of spitting out. The quantity and frequency of spitting reduced significantly. Chewable food was introduced to reinforce the physiological oropharyngeal coordination of swallowing. The patient was advised a home regime of pharyngeal exercises and practice of swallowing with recommended safe consistencies of food, like groups 4 (pudding thick) and 5 (mechanical soft chewable), three to four times a day.

After 17 weeks and 76 sessions of VitalStim therapy, hyolaryngeal excursion became approximately 80%, which was functionally weak (MMT) [14], and the patient was able to swallow foods of groups 3 (honey thick liquids), 4 (pudding thick), 5 (mechanical soft chewable), and 6 (chewy food) slowly and comfortably without any signs of laryngeal penetration. On completion of 79 sessions of treatment in a period of 18 weeks, the patient attained fully functional (MMT) [14] hyolaryngeal excursion and started swallowing foods of groups 1 (thin liquids), 2 (nectar thick liquids), 7 (food that falls apart), and 8 (mixed textures) without any clinical signs and symptoms of laryngeal penetration and aspiration.

It was noted that the patient was able to swallow all consistencies (groups 1 to 8) in a safe manner and rated level 7 on ASHA scale. He was suggested to continue Shaker and Mendelssohn's maneuver for pharyngeal strengthening as well as follow the general aspiration precautions. The NGT was subsequently removed. The patient had a followup in the department of physiotherapy and rehabilitation after 6 months. Reassessment showed no deficit in swallowing function.

## 6. Discussion

Evidence of combined traditional therapy and VitalStim therapy to treat dysphagia due to stroke is documented [6, 12]. Numerous researches exist on dysphagia management in patients with cerebral stroke, but less work has been reported worldwide on the management of dysphagia in brainstem stroke, especially in cases of lateral medullary infarct. One of the case studies on dysphagia following lateral medullary infarct reported resolution of dysphagia 18 months after stroke [2]. The peculiarity of this case was that the patient did not receive any kind of attention to swallowing or intervention until after a year. Also, it signifies that the chances of spontaneous recovery of dysphagia followed by lateral medullary infarct are less compared to dysphagia following a hemispheric stroke [1]. After almost 16 months of dysphagia, the patient started intensive swallow rehabilitation therapy under the guidance of a dysphagia therapist at Max Healthcare, Saket, New Delhi, India, and, in only four months, regardless of severe dysphagia and late intervention, the patient recovered and achieved complete ability to swallow, eat, and drink by mouth.

Technologic advances like VitalStim therapy, transcranial magnetic stimulation (TMS), and functional magnetic stimulation (FMS) have enhanced the assessment and treatment of patients with dysphagia by permitting better quantification of impairment and treatment effectiveness [18]. Dysphagia due to brainstem stroke can be managed efficiently and early intervention may help in reducing recovery period and dysphagia related complications. An important aspect in patient care is improving the recognition and management of dysphagia or the difficulty or inability to swallow.

Fiberoptic endoscopic evaluation of swallowing (FEES) is a useful tool in the assessment of swallowing. The purpose of the examination is to determine if there is aspiration (food or liquid going into the airway) during or after swallowing, or if the food or liquid remains in the throat after the swallowing. It provides information regarding the structure and functions of the pharyngeal phase of swallowing and swallowing safety [19].

The patient's refusal for undergoing fiberoptic endoscopic evaluation of swallowing (FEES) has been the limitation of this study.

Future study is needed in a larger population of patients with lateral medullary stroke to understand the recovery pattern and effectiveness of combined traditional therapy and VitalStim therapy for improving swallowing function.
